# Medication-Related Osteonecrosis of the Jaw in a Patient With Prostate Cancer: A Case Presentation With a One-Month Follow-Up

**DOI:** 10.7759/cureus.110302

**Published:** 2026-06-05

**Authors:** Charbel F Kachouh, Adam M Aboukhalil, Rosine H Hajj Chehadeh, Robert S Daccache, Hiam Y Wehbe, Samar A Bou Jaoude

**Affiliations:** 1 Department of Oral Medicine and Radiology, Saint Joseph University of Beirut, Beirut, LBN; 2 Department of Oral Surgery, Saint Joseph University of Beirut, Beirut, LBN

**Keywords:** bisphosphonate, bisphosphonate-related osteonecrosis of the jaw, fistula, mandibular fractures, osteoporosis, prostate cancer

## Abstract

Medication-related osteonecrosis of the jaw (MRONJ) is an adverse event that most commonly occurs in patients undergoing bisphosphonate therapy and remains a significant challenge for dentists. Its clinical presentation varies according to the stages of the disease, depending on clinical and radiological findings. We report the case of an 81-year-old man presenting with a cutaneous fistula and purulent drainage from the lower right side of the mandible. The patient was under chemotherapy and receiving zoledronic acid treatment, administered as an intravenous injection every six months for 10 years. Oral manifestations of MRONJ can present with diverse lesions requiring careful clinical evaluation, accurate diagnosis, and appropriate management.

## Introduction

Oral lesions are commonly linked to systemic diseases and side effects of medical treatments. Careful recognition, interpretation, and interdisciplinary management are therefore essential for addressing such clinical scenarios, leading to earlier systemic diagnosis, safer therapy, and better overall outcomes [1]. Dentists play a crucial role in detecting early signs of systemic diseases and their manifestations in the oral cavity, extending the impact beyond oral health status [2]. One of the oral manifestations of general conditions is medication-related osteonecrosis of the jaw (MRONJ).

The first cases of MRONJ were reported in the early 2000s. Diagnostic criteria were established by the American Association of Oral and Maxillofacial Surgeons (AAOMS) in 2014 based on the patient’s pharmacological history and clinical and radiographic findings [3]. According to the AAOMS' latest guidelines update in 2022, MRONJ is defined as exposed bone or bone that can be probed through a fistula present in the maxillofacial region, persisting for more than eight weeks in patients treated with antiresorptive or antiangiogenic medications, with no history of radiation therapy or metastatic disease affecting the jaws [4].

MRONJ is further divided into four stages based on clinical presentation. Stage 0 includes patients with nonexposed bone but presenting symptoms or radiographic abnormalities. Stage 1 comprises patients with necrotic and exposed bone or fistulas that probe to bone. Stage 1 patients are usually asymptomatic and show no signs of infection. Stage 2 involves exposed and necrotic bone associated with pain and clinical evidence of infection. Stage 3 includes stage 2 patients with exacerbated clinical presentations such as pathological fracture, extraoral fistulas, or osteolysis extending to the inferior border of the mandible or the sinus floor [4].

Antiresorptive medications include bisphosphonates, receptor activator of nuclear factor kappa-B ligand (RANK-L) inhibitors, and antiangiogenic drugs. Bisphosphonates such as alendronate (Fosamax^®^), ibandronate (Bonviva^®^), and zoledronic acid (Zometa^®^) inhibit bone resorption primarily by targeting osteoclast activity [5]. The Food and Drug Administration has approved these medications for the treatment of conditions characterized by increased bone turnover, including osteoporosis in men and women, Paget’s disease of bone, and malignancy-associated hypercalcemia [6]. Patients receiving bisphosphonates for oncologic indications have a significantly higher risk of developing MRONJ than those treated for osteoporosis or Paget’s disease primarily due to differences in dosage, administration route, and underlying disease factors [7].

This report describes the case of an 81-year-old patient who attended the Saint Joseph University Dental Care Center in Beirut. Clinical examination revealed halitosis, paresthesia of the lip, purulent drainage from the lower right mandible, and a cutaneous fistula on the ipsilateral cheek. Through this case, we aim to provide a concise review of the literature and to highlight the clinical and radiological features of MRONJ, emphasizing the importance of clinicians’ awareness of atypical presentations that may pose diagnostic challenges.

## Case presentation

An 81-year-old male patient was referred by his general dentist to the Oral Medicine and Radiology Department at the Faculty of Dental Medicine, Saint Joseph University of Beirut, for evaluation of a persistent cutaneous fistula in the right mandibular region. The patient signed an informed consent for the use of his clinical and radiographic data for research purposes. The patient’s medical history was significant for prostate cancer, treated with six cycles of chemotherapy completed in 2020, and long-term antiresorptive therapy consisting of intravenous (IV) injections of zoledronic acid (Zometa^®^). The patient also reported a history of thyroid surgery and type 1 diabetes mellitus, although glycated hemoglobin levels had not been monitored for several years. Current medications included antiplatelet therapy (acetylsalicylic acid, Aspicot^®^), antidiabetic treatment (vildagliptin and metformin, Galvus Met R^®^ 50 mg), thyroid hormone replacement (levothyroxine, Euthyrox^®^ 100 µg), statin therapy (rosuvastatin, Rosucor^® ^10 mg), corticosteroids (Prednisone^®^), as well as other supplements and medications (Prostatine® 0.4 mg, Exonar® 40 mg, Neuraxial 150 mg^®^, Vessel Due F^®^, Magnesium B6^®^, Ostrong^®^, Abremia^®^ 250 mg, and V2 Plus^®^). Additionally, the patient reported prior use of a removable partial denture (RPD) for the lower jaw, which was subsequently discontinued due to pain and repeated episodes of soft tissue irritation and gingival trauma associated with its use, especially on the right side of the mandible.

The patient’s chief complaint was a lip paraesthesia, persistent cutaneous fistula located in the right mandibular region, associated with purulent discharge. The patient reported that the lesion has been present for several months and required continuous coverage with a napkin to prevent soiling of clothing. The patient reported that pain was more intense in the evening and, in an attempt to alleviate discomfort, occasionally inserted a heated metal needle into the fistula to facilitate drainage. The patient also reported a persistent foul odor from the lesion as well as halitosis associated with the exposed bone and purulent discharge.

On extraoral examination, facial asymmetry was noted, along with a cutaneous fistula in the right mandibular region exhibiting purulent discharge. The patient also reported paraesthesia involving the right lower lip and chin. Intraoral examination revealed an area of exposed necrotic bone located along the external oblique ridge of the right mandible, rather than the alveolar crest (Figure 1A). The surrounding mucosa showed signs of inflammation. A whitish material adherent to the exposed bone was identified and later determined to be glass ionomer cement (GIC) previously placed by a dentist in an attempt to seal the fistula. A fistulous tract with purulent exudate was also observed (Figures 1B, 1C).

**Figure 1:**
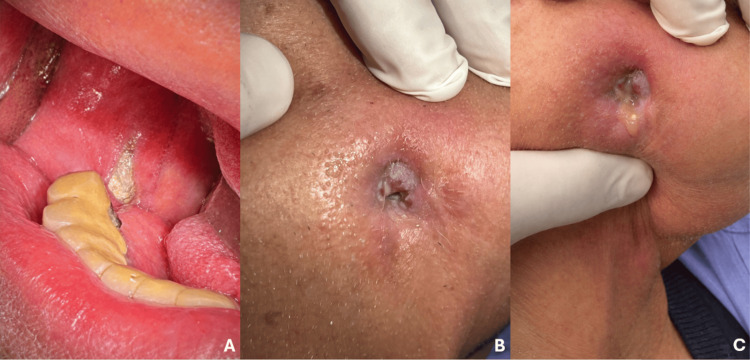
(A) Intraoral presentation. (B) Extraoral presentation of the cutaneous fistula on the lower right side of the mandible. (C) Purulent discharge upon pressure on the right cheek

Panoramic radiograph interpretation

Panoramic radiography revealed multiple findings. In the right maxillary region, one implant was supporting a prosthetic restoration,while a second implant showed peri-implant bone loss and lacked any prosthetic rehabilitation. On the left maxillary side, two endodontically treated roots were supporting a two-unit bridge. In the mandible, an anterior bridge extending from canine to canine was observed, serving as support for the RPD. On the right mandibular side, a 13 x 16 mm radiopaque mass corresponding to previously placed GIC was identified between two separated mandibular fragments. A fracture line was clearly visible traversing the GIC mass, resulting in separation of the mandibular segments (Figure 2).

**Figure 2:**
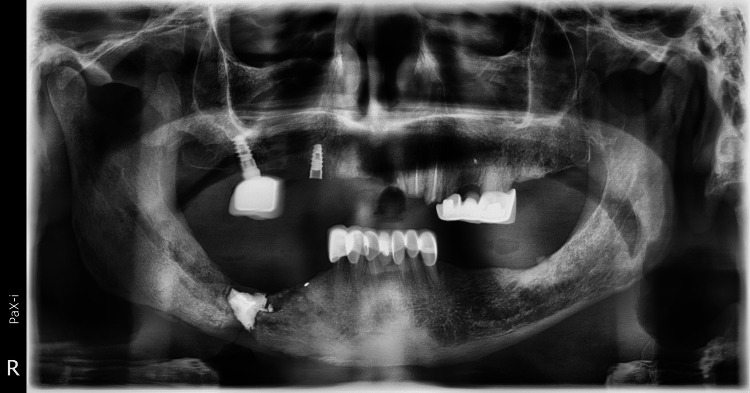
Preoperative panoramic radiograph showing a mandibular fracture line associated with a radiopaque mass on the right side of the mandible

CBCT findings

Cone beam computed tomography (CBCT) imaging revealed extensive pathological changes involving the right mandibular body, characterized by a mixed osteolytic-sclerotic pattern with marked disruption of the normal bone architecture. Sagittal sections confirmed the presence of a pathological fracture, with irregular and poorly defined bone margins, as well as areas suggestive of sequestrum formation. The radiopaque GIC mass was again visualized within the fracture gap. Additionally, a hypodense tract extending inferiorly toward the cutaneous surface was identified, consistent with the course of the cutaneous fistula (Figure 3A).

**Figure 3:**
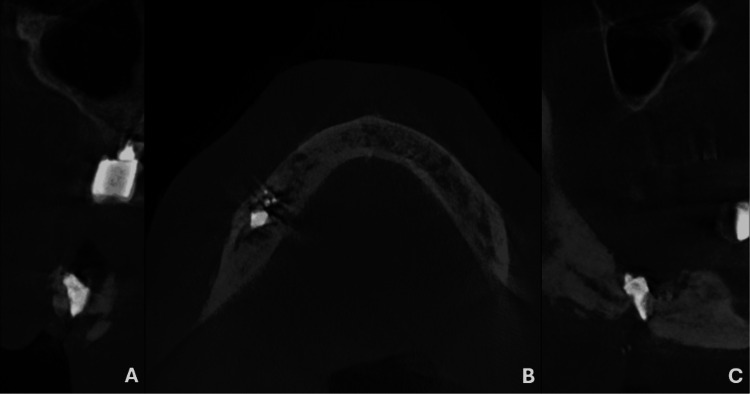
(A) Sagittal CBCT cut. (B) Axial CBCT cut. (C) Tangential CBCT cut CBCT: cone beam computed tomography

Axial sections demonstrated significant osteolysis, with loss of normal trabecular organization and clear discontinuity of both the buccal and lingual cortical plates. A complete fracture line was identified, separating the mandibular body into two distinct segments. A well-defined hyperdense radiopaque mass, corresponding to the previously placed GIC, was observed interposed between the fractured segments (Figure 3B).

Tangential sections showed vertical extension of bone destruction from the alveolar ridge toward the inferior border of the mandible, with cortical perforation on both alveolar and basilar aspects. The fracture line was clearly visible, extending through the GIC material. Importantly, involvement of the inferior alveolar canal was observed, with loss of canal cortication and disruption along its course, correlating with the patient’s reported paraesthesia of the lower lip and chin (Figure 3C). Overall, the CBCT findings were consistent with advanced MRONJ, demonstrating extensive bone destruction, cortical disruption, inferior alveolar canal involvement, fistulous tract formation, and pathological mandibular fracture. Following the AAOMS classification and based on the clinical and radiographic findings, with exposed necrotic bone, purulent discharge, cutaneous fistula, sensory disturbance, and mandibular fracture, the patient was diagnosed with Stage 3 MRONJ [4].

Patient management

Initial measures included patient education, reinforcement of oral hygiene, and avoidance of further local trauma, particularly from the RPD. The patient was also advised to discontinue any self-manipulation of the lesion. Definitive management was to be determined by the maxillofacial surgery team, with consideration of surgical intervention in accordance with current guidelines for Stage 3 MRONJ. Given the advanced stage of the lesion, characterized by extensive osteonecrosis, cutaneous fistulization, and pathological mandibular fracture, the patient was referred to a maxillofacial surgeon for specialized management. Following intensive antibiotic therapy consisting of amoxicillin/clavulanic acid (Augmentin^®^) 2 g daily for two weeks before the surgery, surgical exploration of the right mandibular region was performed, with debridement of the infected site, excision of the cutaneous fistula, and removal of the radiopaque foreign material corresponding to the previously placed GIC.

Following removal of the foreign body, superficial necrotic bone was eliminated by conservative osteotomy until viable bleeding bone margins were obtained. The pathological mandibular fracture was then stabilized by open reduction and internal fixation using a reconstruction plate secured with four screws on each mandibular fragment. Postoperative panoramic radiography confirmed complete removal of the radiopaque mass. The fixation hardware was clearly visible, with the plate and screws maintaining alignment of the mandibular segments. The fracture line remained perceptible radiographically, as expected in the immediate postoperative phase. Interruption of the mandibular canal continuity was also observed in the affected region, consistent with the preoperative inferior alveolar nerve involvement (Figure 4).

**Figure 4:**
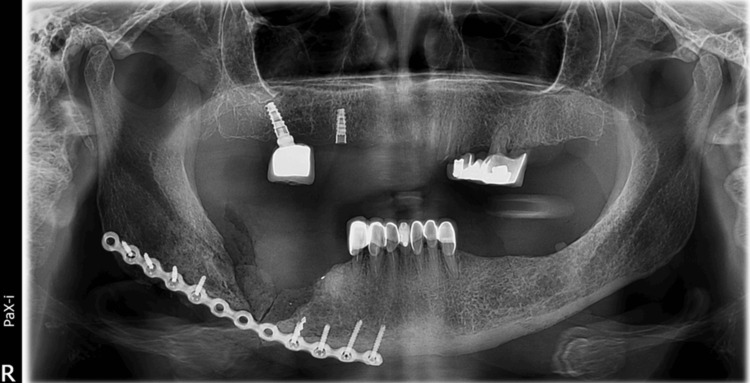
Postoperative panoramic radiograph showing surgical debridement of the lesion site and reconstruction with a fixation plate and screws

The postoperative period was satisfactory. The patient was prescribed antibiotic therapy with amoxicillin/clavulanic acid (Augmentin^®^) at a dose of 2 g/day, administered as 1 g twice daily (morning and evening), for two weeks postoperatively. Postoperative pain management consisted of paracetamol/codeine (Solpadeine^®^) 1 g every six hours as needed. In addition, the patient was instructed to rinse twice daily with 0.12% chlorhexidine mouthwash during the postoperative healing period.

At the one-month follow-up, the lesion was in the healing phase, with a noticeable reduction in its radiological size. Intraoral examination revealed persistent bone exposure (Figure 5A). Extra-orally, following fistula tract excision during the surgery, only a scar was visible with absence of any purulent discharge (Figure 5B). Paresthesia of the lower lip remained present at this follow-up visit. The patient was advised to continue rinsing with 0.12% chlorhexidine mouthwash and warm saline solution, alternating between both solutions regularly. Additionally, vitamin B1, B6, and B12 supplementation was prescribed to the patient, as these vitamins have been reported to exert beneficial effects on nerve injury recovery.

**Figure 5:**
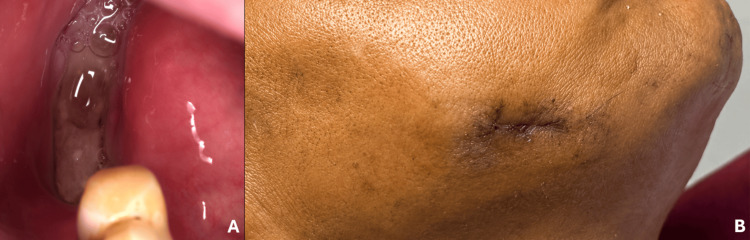
(A) Intraoral healing at one month postoperatively. (B) Extraoral appearance of the scar one month after fistula tract excision. No clinical evidence of purulent discharge or extraoral drainage was observed

## Discussion

MRONJ is associated with several classes of medications, including bisphosphonates (e.g., zoledronic acid, alendronate), RANK-L inhibitors (e.g., denosumab), and antiangiogenic agents (e.g., bevacizumab, sunitinib). These drugs differ in their mechanisms of action but have all been implicated in the development of MRONJ. The patient presented in this case report had been receiving zoledronic acid, a nitrogen-containing bisphosphonate commonly prescribed for the management of skeletal-related complications associated with malignant diseases.

Bisphosphonates, widely used in the management of bone disorders, exert their effects by inhibiting bone resorption by inhibiting osteoclast activity [8]. These bisphosphonates may be administered either orally (per os) or IV, depending on the therapeutic regimen and the patient’s general condition. Despite their efficacy, their use is associated with upper gastrointestinal adverse effects, including dysphagia, nausea, vomiting, and dyspepsia [9]. However, the use of these agents is associated with a significant adverse effect, particularly relevant in dental practice, MRONJ [4].

Several studies have shown that oral bisphosphonates, widely used in the management of osteoporosis and osteopenia, show moderate potency. The risk of MRONJ associated with these agents is low and typically occurs after prolonged exposure, exceeding three years. When MRONJ develops in this context, it is usually localized and characterized by a slow progression. The main triggering factors include dental extractions and poor oral hygiene. Preventive strategies rely on conducting a comprehensive dental assessment before initiating treatment and on avoiding invasive dental procedures during therapy. In contrast, intravenously administered bisphosphonates, such as zoledronic acid, demonstrate significantly greater potency than subcutaneously administered agents, such as denosumab (Prolia^®^). These IV formulations are associated with a markedly higher risk of MRONJ than oral agents, with onset potentially within a few months. In this setting, jaw necrosis tends to be more aggressive and progresses more rapidly. Although the triggering factors are similar to those observed with oral bisphosphonates, their impact is amplified. Consequently, preventive measures are based on the same principles but require more rigorous implementation due to the increased level of risk [10].

Furthermore, this patient, diagnosed with prostate cancer and receiving monthly IV bisphosphonate therapy, has been wearing an RPD for the past 10 years. Continuous trauma from the partial denture, together with bisphosphonate therapy, contributed to the development of mucosal ulceration and underlying bone damage, which subsequently progressed to fistulization and the formation of an orocutaneous communication. This fistula was associated with chronic purulent discharge as well as paresthesia of the right lower lip. However, no extensive bone exposure was observed within the oral cavity. Advanced stages of osteonecrosis of the jaw associated with bisphosphonate therapy may present with pathological mandibular fractures and the formation of cutaneous fistulas, a clinical feature frequently reported in the literature as characteristic of Stage 3 MRONJ. An intense periosteal reaction has also often been observed. Imaging findings, together with histopathological analysis, confirm cortical bone destruction and the progression of the necrotic process [11,12].

In addition, MRONJ most commonly occurs following invasive dental procedures, such as tooth extractions, oral trauma, or infections, particularly in the maxilla and mandible, where these agents tend to accumulate [13]. It is characterized by exposed necrotic bone in the jaws persisting for more than eight weeks in patients undergoing bisphosphonate therapy; the affected bone is clinically evident and radiographically identifiable [14], which aligns with our patient's presentation. In clinical practice, MRONJ presents with variable symptomatology, the severity of which may differ between cases. The most observed clinical signs include jaw pain, tooth mobility, exposed bone in the maxilla or mandible, soft-tissue lesions or ulcerations, hypoesthesia of the lower lip, non-healing post-extraction sockets, and swelling or inflammation of the gingival and osseous tissues [15]. As our patient was under antiresorptive medications for oncologic treatment, particular attention must be given during dental management. Several factors, including the duration of the ongoing bisphosphonate therapy and the presence of concomitant treatments, must be considered [16].

Several studies have identified the use of dental prostheses as a potential risk factor in the development of MRONJ, with this risk primarily attributed to prosthesis-induced trauma [17]. Conversely, other studies suggest that prosthetic management does not pose a significant risk, particularly when compared with invasive oral surgical procedures such as tooth extractions and implant placement. These studies further propose that patients with MRONJ may benefit from prosthetic rehabilitation for both functional and esthetic purposes. It is also important to note that removable dentures are more likely to cause mucosal trauma than fixed prostheses [18].

Significantly, corticosteroid use constitutes a major systemic risk factor for the development of osteonecrosis. Patients receiving bisphosphonates in combination with concomitant corticosteroid therapy have been shown to exhibit higher rates of jaw complications, particularly osteonecrosis and delayed wound healing. Furthermore, diabetes mellitus has also been identified as an aggravating systemic factor. By impairing vascularization and bone healing mechanisms, this condition increases susceptibility to bisphosphonate-related osteonecrosis of the jaw [19].

In this context, regular follow-up with a dental practitioner is essential for patients wearing dentures. Any discomfort or irritation should be reported promptly to allow timely adjustment or replacement of ill-fitting devices, thereby minimizing the risk of soft-tissue injury [18]. Management of MRONJ, particularly in advanced stages (starting from Stage 2), relies mainly on surgical intervention, which is considered the standard approach in many cases. Surgical treatment includes debridement, curettage, sequestrectomy, and resection with or without microvascular reconstruction [19]. Other alternatives, such as hyperbaric oxygen therapy, ozone therapy, and low-level laser therapy, have also been suggested as supportive approaches in the management of MRONJ [4,20].

MRONJ is of particular clinical importance in dental practice due to its association with patients receiving antiresorptive or antiangiogenic therapies. Dental extractions, invasive dentoalveolar procedures, and trauma from ill-fitting removable partial or complete dentures are recognized precipitating factors, emphasizing the essential role of dentists in prevention, early detection, and management. The condition, although relatively uncommon, has a significant impact on patient quality of life and presents diagnostic and therapeutic challenges. Its pathophysiology is multifactorial and is mainly related to suppression of osteoclastic activity and bone remodeling, resulting in impaired bone turnover and reduced healing capacity. Therefore, increased awareness of its prevalence, clinical significance, and underlying biological mechanisms improves diagnostic accuracy and patient outcomes.

## Conclusions

This case highlights a severe presentation of MRONJ associated with long-term bisphosphonate therapy in a medically compromised patient. The progression from prosthesis-induced mucosal trauma to bone exposure, cutaneous fistulization, and pathological mandibular fracture underscores the multifactorial nature of MRONJ. Contributing factors in this case included systemic conditions such as diabetes and corticosteroid use, as well as local factors including chronic irritation and inadequate prosthetic adaptation. This report emphasizes the importance of preventive dental care, regular monitoring, and early management of oral lesions in patients receiving antiresorptive therapy. Particular attention should be given to removable prostheses to avoid mucosal trauma. Early intervention may help prevent progression to advanced stages requiring complex surgical management.
